# Recurrence-free survival as a surrogate endpoint for overall survival after neoadjuvant chemotherapy and surgery for oesophageal squamous cell carcinoma

**DOI:** 10.1093/bjs/znae038

**Published:** 2024-02-20

**Authors:** Jun Okui, Kengo Nagashima, Satoru Matsuda, Yasunori Sato, Akihiko Okamura, Hirofumi Kawakubo, Manabu Muto, Yoshihiro Kakeji, Koji Kono, Hiroya Takeuchi, Masayuki Watanabe, Yuichiro Doki, Takeo Bamba, Takashi Fukuda, Hitoshi Fujiwara, Shinsuke Sato, Kazuhiro Noma, Hiroshi Miyata, Takeo Fujita, Yuko Kitagawa

**Affiliations:** Department of Surgery, Keio University School of Medicine, Tokyo, Japan; Department of Preventive Medicine and Public Health, Keio University School of Medicine, Tokyo, Japan; Biostatistics Unit, Clinical and Translational Research Centre, Keio University Hospital, Tokyo, Japan; Department of Surgery, Keio University School of Medicine, Tokyo, Japan; Department of Preventive Medicine and Public Health, Keio University School of Medicine, Tokyo, Japan; Department of Gastroenterological Surgery, Cancer Institute Hospital of Japanese Foundation for Cancer Research, Tokyo, Japan; Department of Surgery, Keio University School of Medicine, Tokyo, Japan; Department of Therapeutic Oncology, Graduate School of Medicine, Kyoto University, Kyoto, Japan; Division of Gastrointestinal Surgery, Department of Surgery, Kobe University Graduate School of Medicine, Hyogo, Japan; Department of Gastrointestinal Tract Surgery, Fukushima Medical University, Fukushima, Japan; Department of Surgery, Hamamatsu University School of Medicine, Shizuoka, Japan; Department of Gastroenterological Surgery, Cancer Institute Hospital of Japanese Foundation for Cancer Research, Tokyo, Japan; Department of Gastroenterological Surgery, Osaka University Graduate School of Medicine, Osaka, Japan; Department of Digestive Surgery, Niigata Cancer Centre Hospital, Niigata, Japan; Department of Gastrointestinal Surgery, Saitama Cancer Centre, Saitama, Japan; Division of Digestive Surgery, Department of Surgery, Kyoto Prefectural University of Medicine, Kyoto, Japan; Department of Gastroenterological Surgery, Shizuoka General Hospital, Shizuoka, Japan; Department of Gastroenterological Surgery, Okayama University Graduate School of Medicine, Dentistry, and Pharmaceutical Sciences, Okayama, Japan; Department of Digestive Surgery, Osaka International Cancer Institute, Osaka, Japan; Department of Esophageal Surgery, National Cancer Centre Hospital East, Chiba, Japan; Department of Surgery, Keio University School of Medicine, Tokyo, Japan

## Abstract

**Background:**

Overall survival is considered as one of the most important endpoints of treatment efficacy but often requires long follow-up. This study aimed to determine the validity of recurrence-free survival as a surrogate endpoint for overall survival in patients with surgically resectable advanced oesophageal squamous cell carcinoma (OSCC).

**Methods:**

Patients with OSCC who received neoadjuvant cisplatin and 5-fluorouracil, or docetaxel, cisplatin and 5-fluorouracil, at 58 Japanese oesophageal centres certified by the Japan Esophageal Society were reviewed retrospectively. The correlation between recurrence-free and overall survival was assessed using Kendall's τ.

**Results:**

The study included 3154 patients. The 5-year overall and recurrence-free survival rates were 56.6 and 47.7% respectively. The primary analysis revealed a strong correlation between recurrence-free and overall survival (Kendall's τ 0.797, 95% c.i. 0.782 to 0.812) at the individual level. Subgroup analysis showed a positive relationship between a more favourable pathological response to neoadjuvant chemotherapy and a higher τ value. In the meta-regression model, the adjusted *R*^2^ value at the institutional level was 100 (95% c.i. 40.2 to 100)%. The surrogate threshold effect was 0.703.

**Conclusion:**

There was a strong correlation between recurrence-free and overall survival in patients with surgically resectable OSCC who underwent neoadjuvant chemotherapy, and this was more pronounced in patients with a better response to neoadjuvant chemotherapy.

## Teaser

In this retrospective cohort study involving 3154 patients, a strong correlation between recurrence-free and overall survival was established, and this correlation was more pronounced in patients who had a more effective response to neoadjuvant chemotherapy. These findings are beneficial for expediting the development of novel neoadjuvant treatments by shortening the duration of clinical trials.

## Introduction

Worldwide, oesophageal cancer ranks seventh and sixth in cancer incidence and mortality respectively^1^. As oesophageal cancer metastasizes already at early disease stage^2,3^, multimodal treatment is required^4,5^. In Western countries, preoperative chemoradiotherapy or perioperative chemotherapy in combination with transthoracic oesophagectomy is standard of care^6,7^. In Japan, neoadjuvant chemotherapy followed by oesophagectomy with radical lymph node dissection is indicated for oesophageal squamous cell carcinoma (OSCC)^8^. After treatment with curative intent, the risk of recurrence remains high. Hence, adjuvant systemic therapies including the role of immune checkpoint inhibitors are currently being investigated^9,10^.

Overall survival (OS) is considered as one of the most important endpoints of treatment efficacy in RCTs. A disadvantage of using OS as the primary endpoint is that it often requires a long follow-up time and large trial populations to detect statistically significant and clinically meaningful differences between study arms. Another disadvantage is that it is potentially affected by non-cancer causes of death and advances in treatment of recurrent or advanced disease. Therefore, statistically appropriate and clinically relevant surrogate endpoints should be explored.

Disease-free survival (DFS), progression-free survival (PFS), and recurrence-free survival (RFS) have been investigated in colorectal, breast, lung, and gastric cancers^11–14^. However, few studies have examined the use of these endpoints in oesophageal cancer. Kataoka *et al*.^15^ showed that PFS was not an appropriate surrogate endpoint for OS using data from 10 clinical trials in oesophageal cancer. On the contrary, Ajani *et al*.^16^ demonstrated in a literature-based study that the HRs for DFS and PFS correlated with those for OS, and concluded that both reflect OS. However, both studies used aggregated data such as HRs and did not analyse individual-patient data. Studies using individual-patient data are more robust because trial-level correlations and individual-level correlations may not be consistent^17^.

This primary aim of this study was to evaluate RFS as a surrogate endpoint for OS in patients who underwent surgery after neoadjuvant chemotherapy for OSCC. The impact of pCR rate on the association between RFS and OS was also assessed.

## Methods

### Study design

This retrospective, multicentre observational study was conducted across 58 Japanese hospitals recognized by the Authorized Institute for Board Certified Esophageal Surgeons by the Japan Esophageal Society. The study was approved by Keio University School of Medicine Ethics Committee and by all the participating centres (Ethics Approval Number 20231069). The study was performed in accordance with the Declaration of Helsinki. The need for informed consent was waived owing to the retrospective nature of the study. This study adhered to the STROBE guidelines^18^.

### Patient selection

The study included patients with OSCC who underwent subtotal oesophagectomy between 2010 and 2015. Patients with clinical stage I, II, III (excluding cT1 N0 and cT4b) or IV OSCC based on supraclavicular lymph node metastases, and who underwent neoadjuvant chemotherapy with DCF (docetaxel, cisplatin, and 5-fluorouracil (5-FU)) or CF (cisplatin plus 5-FU) were included. Patients undergoing salvage oesophagectomy after definitive chemoradiotherapy were excluded.

### Data collection and definitions

Information on patient characteristics, clinicopathological factors, and surgical procedures was collected retrospectively from each hospital. Clinical stage before treatment was determined via oesophagogastroduodenoscopy (OGD) and CT. Based on the data, the extent of tumour spread was reassessed using the eighth edition of the TNM classification established by the UICC^19^. Primary tumours were examined to evaluate the histological response to preoperative treatment in accordance with the Japanese Classification of Esophageal Cancer^20,21^. This classification scheme includes five grades: grade 0, no tumour response; grade 1a, necrotic or fibrotic change observed in less than one-third of the tumour; grade 1b, necrotic or fibrotic change observed in between one- and two-thirds of the tumour; grade 2, more than two-thirds of the tumour is necrotic or fibrotic; and grade 3, no viable tumour cells.

### Treatment

Surgery entailed a transthoracic oesophagectomy with right thoracotomy and gastric tube reconstruction via the posterior mediastinal or retrosternal route and a two- or three-field lymph node dissection^22,23^. This has been the standard curative surgical procedure since before 2010 in Japan. Mediastinal lymph nodes with bilateral recurrent nerve and abdominal lymph nodes were dissected routinely, including the paracardial lymph nodes and lymph nodes along the lesser curvature and left gastric artery. Additionally, supraclavicular lymph node dissection was performed if the primary tumour was situated between the upper and mid-thoracic oesophagus. All patients with stage IVB disease had supraclavicular lymph node metastases and underwent three-field lymph node dissection as recommended for these patients^20,24^. Two courses of CF chemotherapy every 3 weeks was the standard preoperative treatment at most centres, in accordance with the JCOG 9907 study in Japan^8^. Treatment with three courses of DCF was an alternative treatment option mostly administered every 3 weeks^25^.

Postoperative follow-up included OGD and CT every 4–6 months annually until 5 years after operation.

### Statistical analysis

OS was calculated from the date of surgery until day of death or last follow-up. RFS was calculated from the date of surgery until the day of death, recurrence, or last follow-up. At the individual level, Kendall's *τ* was employed to assess surrogacy between RFS and OS. Kendall's *τ* is a rank correlation coefficient ranging from −1 to 1, with values closer to 1 indicating a higher correlation. The association between the true endpoint (OS) and the surrogate endpoint (RFS) was evaluated using the following four methods. As a primary analysis, the illness–death model-based method^26^ was used to estimate Kendall's *τ* between RFS and OS, taking into account the effects of competing risks. Simulation results showed that the illness-death model-based method performed well across several scenarios^26^. The survival process in patients with cancer can be represented using a three-state illness–death model, with states corresponding to before recurrence, recurrence, and death. Estimating the correlation between the two failure time endpoints involved modelling the transition intensities between these states. As secondary analyses, the two-step method was used (a bivariable model based on the Clayton copula combined with the trial-specific Weibull model)^27^, the joint frailty–copula model based on the Clayton copula^28^, and the non-parametric inverse probability of censoring weighting (IPCW) method^29^. The former two methods measure dependence structures between RFS and OS using copula models, which are used widely for modelling failure time endpoints. The IPCW method is an extension of Kendall’s τ estimation method for survival time outcomes that accounts for the probability of censoring. As a subgroup analysis, Kendall's *τ* was estimated using the illness–death model-based method for each pathological grade (0, 1a, 1b, 2, 3), and for patients who received adjuvant chemotherapy and those who did not. As an exploratory analysis, surrogacy between each short-term postoperative endpoint (pCR or pathological grade) and OS was investigated using novel statistical methods. *τ* was estimated using a modified IPCW estimator to uncensored binary variables^29^. The modified method adjusts for tie data that occur when the IPCW method is applied to a survival time outcome and a categorical outcome. Moreover, *C*-index was estimated to examine the ability of pCR or pathological grade to discriminate OS^30^.

At the institutional level, the coefficient of determination, *R*^2^, between the natural logarithm of the age-adjusted HRs for RFS and OS was used to assess surrogacy. HRs were calculated using DCF-treated patients as the treatment group and CF-treated patients as the control group. For estimating adjusted *R*^2^, a meta-regression model was used that accounted for the sample size and HR variability across hospitals by using the generic inverse-variance method^31^. Furthermore, the surrogate threshold effect (STE) was estimated, indicating the minimum RFS treatment effect required to predict a non-zero effect on OS^32^. In future trials, to predict a non-zero OS effect, the upper limit of the prediction interval for the estimated HR for RFS should be lower than the STE.

R version 4.3.0 (R Foundation for Statistical Computing, Vienna, Austria) was used, and the packages surrosurv, joint.Cox, metagen, metafor, dynpred, and survC1 were employed to implement the methods described above^27,28,31,33,34^. The 95% confidence interval for *τ* was obtained by the surrosurv package and the 95% confidence interval for *R*^2^ was estimated by the bootstrap method.

## Results

In total, 3154 patients were included. *Figure S1* shows the study flow chart and patient characteristics are summarized in *Table 1*. The mean(s.d.) age was 65.45(7.80) years and half of the patients had a tumour in the mid-thoracic oesophagus. One Thousand Forty-Six (33.2%) patients received neoadjuvant DCF chemotherapy, and adjuvant therapy was given to 398 patients (13.2%). Clinical and pathological disease stages are shown in *Table 1*.

**Table 1:** Patient characteristics

	No. of patients* (*n* = 3154)
**Age (years), mean(s.d.)**	65.45(7.80)
**Sex**	
F	480 (15.2)
M	2674 (84.8)
**Tumour location**	
Cervical	58 (1.8)
Upper thoracic	401 (12.7)
Mid-thoracic	1566 (49.7)
Lower thoracic	1129 (35.8)
**Clinical tumour category**	
cT1	295 (9.4)
cT2	683 (21.7)
cT3	2149 (68.2)
cT4a	26 (0.8)
**Clinical node category**	
cN0	736 (23.3)
cN1	1575 (49.9)
cN2	734 (23.3)
3	109 (3.5)
**Clinical metastasis category**	
cM0	3014 (95.6)
cM1	140 (4.4)
**Clinical stage**	
I	224 (7.1)
II	1008 (32.0)
III	1664 (52.8)
IVA	117 (3.7)
IVB	140 (4.4)
**BMI (kg/m^2^), mean(s.d.)**	21.30(3.09)
**Chemotherapy regimen**	
CF	2108 (66.8)
DCF	1046 (33.2)
**Surgical approach**	
Minimally invasive	1625 (51.6)
Open	1525 (48.4)
**Pathological tumour category**	
pT0	199 (6.4)
pT1	792 (25.6)
pT2	496 (16.0)
pT3	1523 (49.2)
pT4a	62 (2.0)
pT4b	22 (0.7)
**Pathological node category**	
pN0	1142 (36.3)
pN1	988 (31.4)
pN2	702 (22.3)
pN3	313 (10.0)
**Pathological metastasis category**	
pM0	2886 (92.5)
pM1	235 (7.5)
**Pathological stage**	
0	156 (5.0)
I	352 (11.3)
II	914 (29.4)
III	1185 (38.2)
IVA	264 (8.5)
IVB	235 (7.6)
**Pathological grade**	
0	269 (9.0)
1a	1567 (52.5)
1b	458 (15.3)
2	493 (16.5)
3	197 (6.6)
**pCR**	
pCR	145 (4.9)
non-pCR	2799 (95.1)
**Pathological resection status**	
R0	2857 (94.4)
R1	170 (5.6)
**Adjuvant therapy**	398 (13.2)

*Values are *n* (%) unless otherwise indicated. CF, cisplatin + 5-fluorouracil; DCF, docetaxel + cisplatin + 5-fluorouracil.

**Fig. 1:**
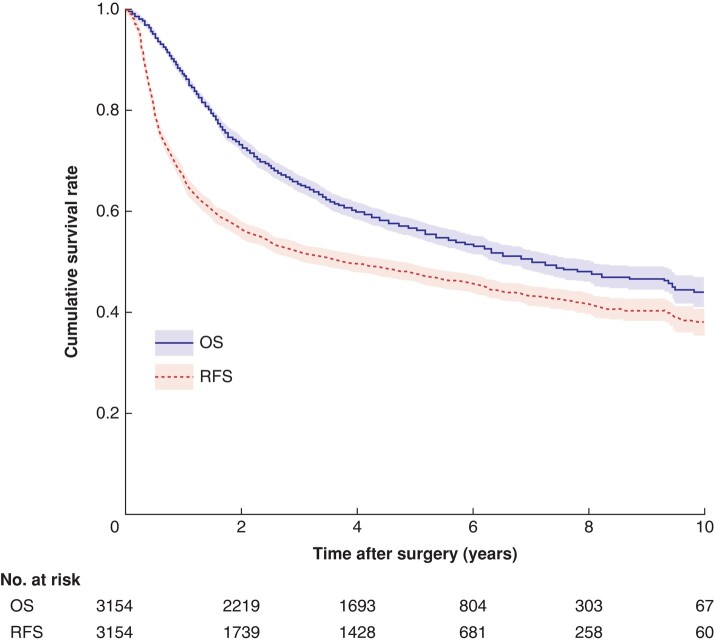
Kaplan–Meier estimates of overall and recurrence-free survival Shaded areas represent 95% confidence intervals. OS, overall survival; RFS, recurrence-free survival.

**Fig. 2:**
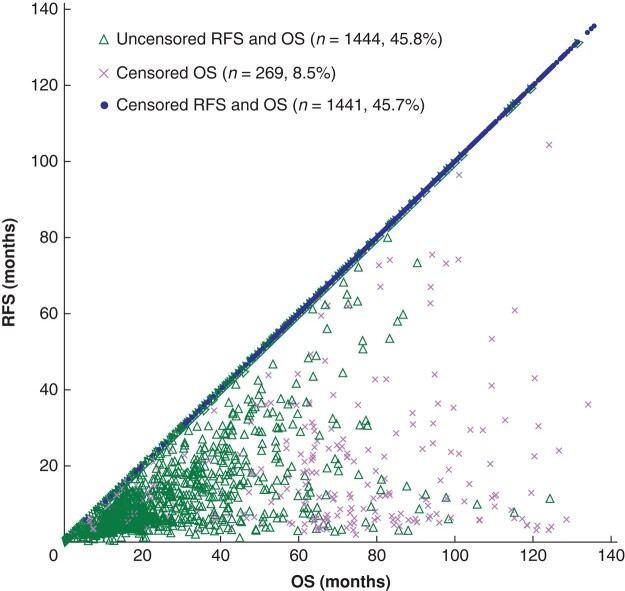
Scatter plot of bivariable survival times OS, overall survival; RFS, recurrence-free survival.

The 5-year OS and RFS rates were 56.6 (95% c.i. 54.9 to 58.4) and 47.7 (46.0 to 49.5)% respectively (*Fig. 1*). There were 1713 RFS events, 1444 deaths, and 1441 patients with both OS and RFS censored (*Fig. 2*). In the primary analysis using the illness–death model-based method at the individual level, a strong correlation between RFS and OS was found (Kendall's τ 0.797, 95% c.i. 0.782 to 0.812) (*Table 2*). Among the statistical analysis methods, the highest τ value of 0.805 (0.791 to 0.818) was obtained with the IPCW method.

**Table 2:** Kendall's τ values for primary and secondary analyses

	Method	Kendall's τ
RFS	Illness–death model	0.797 (0.782, 0.812)
	Two-step copula-based model (Clayton)	0.746 (0.725, 0.766)
	Joint frailty–copula model	0.767 (0.752, 0.783)
	IPCW method	0.805 (0.791, 0.818)
pCR	Modified IPCW method	−0.025 (−0.335, 0.285)
Pathological grade	Modified IPCW method	0.062 (−0.086, 0.209)

Values in parentheses are 95% confidence intervals. The illness–death model-based method is the primary analysis; other methods comprise the secondary analysis. RFS, recurrence-free survival; IPCW, inverse probability of censoring weighting.

Subgroup analysis showed that an increasing τ value corresponded to a more favourable pathological response (*Table 3*). The results demonstrated that OS and RFS were more strongly correlated in patients with a better treatment response to neoadjuvant chemotherapy. Moreover, the proportion of patients with equal OS and RFS and non-cancer deaths among all deaths increased as the pathological grade increased. The τ value was 0.721 for the patients who received adjuvant chemotherapy and 0.808 for those who did not (*Table S1*). In the exploratory analysis, the τ value was −0.025 (−0.335 to 0.285) between pCR and OS, and 0.062 (−0.086 to 0.209) between pathological grade and OS. Similarly, the respective *C*-index values were 0.521 and 0.575 (*Table S2*).

**Table 3:** Kendall's τ between recurrence-free and overall survival: subgroup analysis

Pathological grade	*n*	Patients with equal OS and RFS as a proportion of patients excluding those with both OS and RFS censored	Non-cancer death as a proportion of all causes of death†	Kendall's τ*
0	269	28 of 175 (16.0)	24 of 139 (17.3)	0.732 (0.597, 0.868)
1a	1567	143 of 950 (15.1)	149 of 800 (18.6)	0.749 (0.726, 0.773)
1b	458	56 of 251 (22.3)	51 of 202 (25.2)	0.781 (0.727, 0.836)
2	493	58 of 202 (28.7)	58 of 157 (36.9)	0.846 (0.746, 0.945)
3	197	22 of 52 (42.3)	19 of 39 (48.7)	0.928 (0.441, 1.000)

Values are *n* (%) unless otherwise indicated; *values in parentheses are 95% confidence intervals. †Excluding patients with unknown cause of death. The illness–death model-based method was used. OS, overall survival; RFS, recurrence-free survival.

**Fig. 3:**
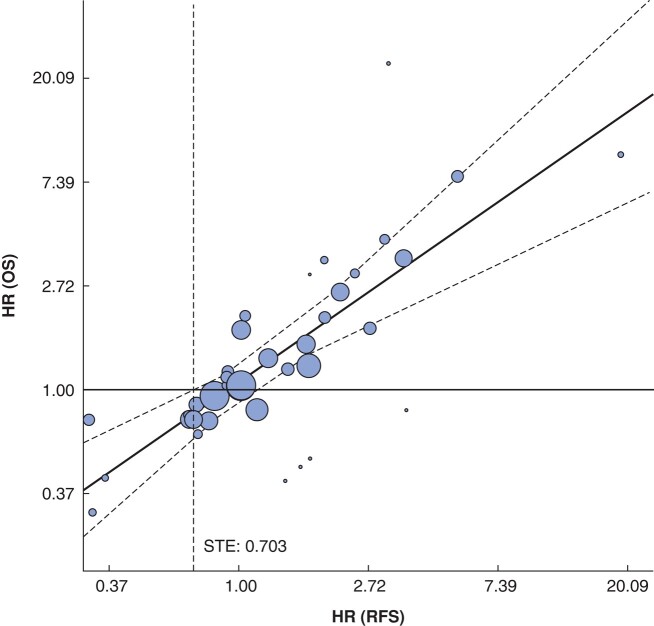
Institutional-level association between treatment effects A log scale was used for both axes. Each institution is represented by a bubble of a size proportional to the sample size and HR variability. The curved and dashed lines represent the 95% prediction limit. The straight dashed line represents STE. The 95% prediction limits illustrate the expected range of OS effects given specific RFS effects. The STE was defined as the point where the upper prediction limit intersected the horizontal line indicating a hazard ratio of 1 for OS (null hypothesis). OS, overall survival; RFS, recurrence-free survival; STE, surrogate threshold effect. Equation for meta-regression model: ln(HR_OS_) = 0.868 × ln(HR_RFS_) + 0.070. Adjusted *R*^2^: 100 (95% c.i. 40.2 to 100)%.

At the institutional level, the adjusted *R*^2^ was 100 (95% c.i. 40.2 to 100)%; this was obtained using the meta-regression model adjusted for sample size and variability in HR. The equation for the meta-regression model was ln(HR_OS_) = 0.868 × ln(HR_RFS_) + 0.070. The slope of 0.868 is close to 1, indicating a strong correlation between OS and RFS (*Fig. 3*). The 95% prediction limits illustrate the expected range of OS effects given specific RFS effects. The STE was defined as the point where the upper prediction limit intersected the horizontal line indicating a HR of 1 for OS (null hypothesis). The STE value was 0.703. Therefore, in future trials with treatment settings similar to those of the present study, a HR for RFS below 0.703 would predict a HR for OS below 1 with 95% probability.

## Discussion

The present study has demonstrated a strong correlation between RFS and OS in patients with surgically resectable OSCC. Haslam *et al*.^35^ conducted a large umbrella analysis of surrogate validation studies and reported that most surrogate endpoints in oncology had a low or modest correlation with OS. However, previous studies were solely literature-based and relied on aggregated data, such as HRs. Conducting studies using individual-patient data is crucial because correlations observed at the trial level may not align with those observed at the individual level^17^. To date, there have been few studies on surrogate endpoints using individual-patient data. A study^36^ in China investigated surrogate endpoints in 292 patients with advanced OSCC treated with immunochemotherapy, and concluded that the treatment effects for PFS had a weak correlation with the treatment effects for OS. However, the study had a limited sample size. The finding that RFS is a surrogate endpoint for OS in the present study may have implications for future studies as this may accommodate a shorter follow-up to reach the primary endpoint of the study.

An important finding was that OS and RFS were more strongly correlated in patients who responded better to preoperative chemotherapy. In the RCT data set analysed by Weber *et al*.^26^, the correlation between PFS and OS was stronger in the treatment group than in the control group. They speculated that the possible mechanism could be that intensive therapy reduces the hazard of progression, thereby resulting in an increased proportion of patients with equal PFS and OS, and thus resulting in an increased τ value. This reflects that OS and RFS are congruent when more patients die from non-cancer causes without recurrence. Consistent results were obtained in the present study: the more effective the preoperative chemotherapy, the higher the proportion of non-cancer deaths and the higher the proportion of patients with equal OS and RFS (*Table 3*). These findings indicate that RFS may be used as a surrogate primary outcome in trials of neoadjuvant treatment modalities leading to high pCR rates. However, studies have reported that the prognosis of pCR differs between neoadjuvant chemotherapy and neoadjuvant chemoradiotherapy^37^. Further research is warranted to determine whether RFS can be applied as a treatment endpoint in modalities other than neoadjuvant chemotherapy.

Kendall's τ value was compared between RFS and OS using four statistical methods. Simulation results showed that the illness–death model-based method performed well across several scenarios^26^ and was thus used as the primary method in this study. The τ value for the illness–death model was high among four methods and consistent with the simulation results. Simulation results also showed that the IPCW method performed well even with higher censoring rates^26^. Similarly, the τ value calculated using this method was the highest in the present study. The τ value obtained using the other methods were consistently high, indicating the robustness of the study results.

Unlike the results between RFS and OS, the correlation between short-term endpoints (pCR or pathological grade) and OS showed only low τ and *C*-index values. Petrelli *et al*.^38^ conducted a study using pCR as a surrogate for OS and concluded that there was no correlation between the two endpoints. However, the study calculated the coefficient of determination between the two endpoints using only literature-level ORs and HRs. In the present study, the analysis was robust because a novel statistical method was used to examine the surrogacy of binary variables and OS at the individual level. The data showed an association between these short-term endpoints and OS, but it was not strong enough to discriminate on its own, the *C*-index being slightly higher than 0.5. Furthermore, the τ value of the binary variable has a theoretical upper limit of 0.75; hence, there is also the problem that a high value cannot be obtained. It was reported previously that pathological grade is a reliable predictor of prognosis^39^, and that endoscopic response is correlated with prognosis^40^. In the future, early postoperative outcomes, such as pCR, may be used as the primary endpoint to shorten the duration of clinical trials, such as phase II trials. Further studies using individual-patient data from RCTs are warranted to draw reliable conclusions.

The study has limitations. First, it was retrospective and relied on real-world data, thereby resulting in a substantial proportion of censoring (*Fig. 2*). Although the estimation of Kendall's τ employed an illness–death-based model that accommodated censoring, further evaluation using individual-patient data in RCTs that typically exhibit lower levels of censoring are needed. Second, the STE for the HR of RFS was 0.703, which is generally considered to be a fairly strict criterion. However, the study was a retrospective simulation of the two neoadjuvant therapy approaches within two treatment arms. Consequently, the quantitative assessment should be interpreted with caution. Furthermore, a separate study^41^ comparing the clinical outcomes of CF and of DCF treatments was performed. Finally, only 13.2% of the patients received adjuvant chemotherapy in this study and surrogacy may not be demonstrated in patients who routinely undergo adjuvant therapy. Subgroup analysis was performed exclusively for those having adjuvant chemotherapy; although Kendall's τ value remained high, the results should be interpreted with caution owing to the relatively small sample size. Similarly, immunochemotherapy is emerging in adjuvant and postrelapse treatment and this may require further study.

## Supplementary Material

znae038_Supplementary_Data

## Data Availability

The data are considered personally identifiable, although all personal identifiers have been removed before data-cleansing and analyses. Therefore, the data used in the present study are not publicly available.

## References

[1] Bray F , Ferlay J, Soerjomataram I, Siegel RL, Torre LA, Jemal A. Global cancer statistics 2018: GLOBOCAN estimates of incidence and mortality worldwide for 36 cancers in 185 countries. CA Cancer J Clin 2018;68:394–424 30207593 10.3322/caac.21492

[2] Akutsu Y , Kato K, Igaki H, Ito Y, Nozaki I, Daiko H et al The prevalence of overall and initial lymph node metastases in clinical T1 N0 thoracic esophageal cancer. Ann Surg 2016;264:1009–1015 27420375 10.1097/SLA.0000000000001557

[3] Takeuchi H , Fujii H, Ando N, Ozawa S, Saikawa Y, Suda K et al Validation study of radio-guided sentinel lymph node navigation in esophageal cancer. Ann Surg 2009;249:757–763 19387329 10.1097/SLA.0b013e3181a38e89

[4] Watanabe M , Otake R, Kozuki R, Toihata T, Takahashi K, Okamura A et al Recent progress in multidisciplinary treatment for patients with esophageal cancer. Surg Today 2020;50:12–20 31535225 10.1007/s00595-019-01878-7 PMC6952324

[5] Tsuji T , Matsuda S, Takeuchi M, Kawakubo H, Kitagawa Y. Updates of perioperative multidisciplinary treatment for surgically resectable esophageal cancer. Jpn J Clin Oncol 2023;53:645–652 37282626 10.1093/jjco/hyad051

[6] Lordick F , Mariette C, Haustermans K, Obermannová R, Arnold D. Oesophageal cancer: ESMO clinical practice guidelines for diagnosis, treatment and follow-up. Ann Oncol 2016;27:v50–v57 27664261 10.1093/annonc/mdw329

[7] Ajani JA , D’Amico TA, Bentrem DJ, Chao J, Corvera C, Das P et al Esophageal and esophagogastric junction cancers, version 2.2019, NCCN clinical practice guidelines in oncology. J Natl Compr Canc Netw 2019;17:855–883 31319389 10.6004/jnccn.2019.0033

[8] Ando N , Kato H, Igaki H, Shinoda M, Ozawa S, Shimizu H et al A randomized trial comparing postoperative adjuvant chemotherapy with cisplatin and 5-fluorouracil *versus* preoperative chemotherapy for localized advanced squamous cell carcinoma of the thoracic esophagus (JCOG9907). Ann Surg Oncol 2012;19:68–74 21879261 10.1245/s10434-011-2049-9

[9] Kelly RJ , Ajani JA, Kuzdzal J, Zander T, Van Cutsem E, Piessen G et al Adjuvant nivolumab in resected esophageal or gastroesophageal junction cancer. N Engl J Med 2021;384:1191–1203 33789008 10.1056/NEJMoa2032125

[10] Doki Y , Ajani JA, Kato K, Xu J, Wyrwicz L, Motoyama S et al Nivolumab combination therapy in advanced esophageal squamous-cell carcinoma. N Engl J Med 2022;386:449–462 35108470 10.1056/NEJMoa2111380

[11] Buyse M , Burzykowski T, Michiels S, Carroll K. Individual- and trial-level surrogacy in colorectal cancer. Stat Methods Med Res 2008;17:467–475 18285439 10.1177/0962280207081864

[12] Burzykowski T , Buyse M, Piccart-Gebhart MJ, Sledge G, Carmichael J, Lück HJ et al Evaluation of tumor response, disease control, progression-free survival, and time to progression as potential surrogate end points in metastatic breast cancer. J Clin Oncol 2008;26:1987–1992 18421050 10.1200/JCO.2007.10.8407

[13] Mauguen A , Pignon JP, Burdett S, Domerg C, Fisher D, Paulus R et al Surrogate endpoints for overall survival in chemotherapy and radiotherapy trials in operable and locally advanced lung cancer: a re-analysis of meta-analyses of individual patients’ data. Lancet Oncol 2013;14:619–626 23680111 10.1016/S1470-2045(13)70158-X PMC3732017

[14] Oba K , Paoletti X, Alberts S, Bang YJ, Benedetti J, Bleiberg H et al Disease-free survival as a surrogate for overall survival in adjuvant trials of gastric cancer: a meta-analysis. J Natl Cancer Inst 2013;105:1600–1607 24108812 10.1093/jnci/djt270 PMC4202244

[15] Kataoka K , Nakamura K, Mizusawa J, Kato K, Eba J, Katayama H et al Surrogacy of progression-free survival (PFS) for overall survival (OS) in esophageal cancer trials with preoperative therapy: literature-based meta-analysis. Eur J Surg Oncol 2017;43:1956–1961 28747249 10.1016/j.ejso.2017.06.017

[16] Ajani JA , Leung L, Singh P, Kurt M, Kim I, Pourrahmat MM et al Disease-free survival as a surrogate endpoint for overall survival in adults with resectable esophageal or gastroesophageal junction cancer: a correlation meta-analysis. Eur J Cancer 2022;170:119–130 35605522 10.1016/j.ejca.2022.04.027

[17] Paoletti X , Lewsley LA, Daniele G, Cook A, Yanaihara N, Tinker A et al Assessment of progression-free survival as a surrogate end point of overall survival in first-line treatment of ovarian cancer: a systematic review and meta-analysis. JAMA Netw Open 2020;3:e1918939 31922558 10.1001/jamanetworkopen.2019.18939 PMC6991254

[18] Vandenbroucke JP , von Elm E, Altman DG, Gøtzsche PC, Mulrow CD, Pocock SJ et al Strengthening the reporting of observational studies in epidemiology (STROBE): explanation and elaboration. PLoS Med 2007;4:e297 17941715 10.1371/journal.pmed.0040297 PMC2020496

[19] Rice TW , Patil DT, Blackstone EH. 8th edition AJCC/UICC staging of cancers of the esophagus and esophagogastric junction: application to clinical practice. Ann Cardiothorac Surg 2017;6:119–130 28447000 10.21037/acs.2017.03.14 PMC5387145

[20] Japan Esophageal Society . Japanese classification of esophageal cancer, 11th edition: part I. Esophagus 2017;14:1–36 28111535 10.1007/s10388-016-0551-7 PMC5222932

[21] Japan Esophageal Society . Japanese classification of esophageal cancer, 11th edition: part II and III. Esophagus 2017;14:37–65 28111536 10.1007/s10388-016-0556-2 PMC5222925

[22] Schuring N , van Berge Henegouwen MI, Gisbertz SS. History and evidence for state of the art of lymphadenectomy in esophageal cancer surgery. Dis Esophagus 2023; DOI:10.1093/dote/doad065 [Epub ahead of print] 10.1093/dote/doad065 PMC10987971 38048446

[23] Matsuda S , Takeuchi M, Kawakubo H, Kitagawa Y. Lymph node metastatic patterns and the development of multidisciplinary treatment for esophageal cancer. Dis Esophagus 2023;36:doad006 36857594 10.1093/dote/doad006 PMC10061432

[24] Matsuda S , Takeuchi H, Kawakubo H, Kitagawa Y. Three-field lymph node dissection in esophageal cancer surgery. J Thorac Dis 2017;9:S731–S740 28815069 10.21037/jtd.2017.03.171 PMC5538994

[25] Kato K , Ito Y, Daiko H, Ozawa S, Ogata T, Hara H et al A randomized controlled phase III trial comparing two chemotherapy regimen and chemoradiotherapy regimen as neoadjuvant treatment for locally advanced esophageal cancer, JCOG1109 NExT study. J Clin Orthod 2022;40:238–238

[26] Weber EM , Titman AC. Quantifying the association between progression-free survival and overall survival in oncology trials using Kendall’s τ. Stat Med 2019;38:703–719 30311243 10.1002/sim.8001 PMC6585767

[27] Burzykowski T , Molenberghs G, Buyse M, Geys H, Renard D. Validation of surrogate end points in multiple randomized clinical trials with failure time end points. J R Stat Soc Ser C Appl Stat 2001;50:405–422

[28] Emura T , Nakatochi M, Murotani K, Rondeau V. A joint frailty–copula model between tumour progression and death for meta-analysis. Stat Methods Med Res 2017;26:2649–2666 26384516 10.1177/0962280215604510

[29] Lakhal L , Rivest LP, Beaudoin D. IPCW estimator for Kendall’s tau under bivariate censoring. Int J Biostat 2009;5:1–20 120231866

[30] Uno H , Cai T, Pencina MJ, D’Agostino RB, Wei LJ. On the C-statistics for evaluating overall adequacy of risk prediction procedures with censored survival data. Stat Med 2011;30:1105–1117 21484848 10.1002/sim.4154 PMC3079915

[31] Balduzzi S , Rücker G, Schwarzer G. How to perform a meta-analysis with R: a practical tutorial. Evid Based Ment Health 2019;22:153–160 31563865 10.1136/ebmental-2019-300117 PMC10231495

[32] Burzykowski T , Buyse M. Surrogate threshold effect: an alternative measure for meta-analytic surrogate endpoint validation. Pharm Stat 2006;5:173–186 17080751 10.1002/pst.207

[33] Rotolo F , Paoletti X, Michiels S. Surrosurv: an R package for the evaluation of failure time surrogate endpoints in individual patient data meta-analyses of randomized clinical trials. Comput Methods Programs Biomed 2018;155:189–198 29512498 10.1016/j.cmpb.2017.12.005

[34] Viechtbauer W . Conducting meta-analyses in R with the metafor package. J Stat Softw 2010;36:1–48

[35] Haslam A , Hey SP, Gill J, Prasad V. A systematic review of trial-level meta-analyses measuring the strength of association between surrogate end-points and overall survival in oncology. Eur J Cancer 2019;106:196–211 30528804 10.1016/j.ejca.2018.11.012

[36] Zhang Z , Xie C, Gao T, Yang Y, Yang Y, Zhao L. Identification on surrogating overall survival with progression-free survival of first-line immunochemotherapy in advanced esophageal squamous cell carcinoma—an exploration of surrogate endpoint. BMC Cancer 2023;23:145 36765311 10.1186/s12885-023-10613-y PMC9921746

[37] Cools-Lartigue J , Markar S, Mueller C, Hofstetter W, Nilsson M, Ilonen I et al An international cohort study of prognosis associated with pathologically complete response following neoadjuvant chemotherapy *versus* chemoradiotherapy of surgical treated esophageal adenocarcinoma. Ann Surg 2022;276:799–805 35861351 10.1097/SLA.0000000000005619

[38] Petrelli F , Tomasello G, Barni S. Surrogate end-points for overall survival in 22 neoadjuvant trials of gastro-oesophageal cancers. Eur J Cancer 2017;76:8–16 28262586 10.1016/j.ejca.2017.01.032

[39] Matsuda S , Kitagawa Y, Okui J, Okamura A, Kawakubo H, Takemura R et al Nationwide validation study of the prognostic significance of stratification using pathological stage and response to neoadjuvant chemotherapy for esophageal squamous cell carcinoma. Ann Surg 2023;278:e234–e239 36538635 10.1097/SLA.0000000000005701

[40] Matsuda S , Kitagawa Y, Okui J, Okamura A, Kawakubo H, Takemura R et al Prognostic impact of endoscopic response evaluation after neoadjuvant chemotherapy for esophageal squamous cell carcinoma: a nationwide validation study. Esophagus 2023;20:455–464 36964333 10.1007/s10388-023-00998-x

[41] Matsuda S , Kitagawa Y, Takemura R, Okui J, Okamura A, Kawakubo H et al Real-world evaluation of the efficacy of neoadjuvant DCF over CF in esophageal squamous cell carcinoma: propensity score matched analysis from 85 authorized institutes for esophageal cancer in Japan. Ann Surg 2022;278:e35–e42 35837977 10.1097/SLA.0000000000005533

