# Supplementary material for: Recurrence-free survival as a surrogate endpoint for overall survival after neoadjuvant chemotherapy and surgery for oesophageal squamous cell carcinoma
Source: Br J Surg. 2024 Feb 20;111(2):znae038. doi: 10.1093/bjs/znae038 (PMC10878553; doi:10.1093/bjs/znae038)
Supplement: znae038_Supplementary_Data [file znae038_supplementary_data.docx]

**Article type:** Original article

**Title:** Recurrence-free survival as a surrogate endpoint for overall survival in oesophageal squamous cell carcinoma

Jun Okui, MD, MPH^1,2^, Kengo Nagashima, PhD^3^, Satoru Matsuda, MD, PhD^1,*^, Yasunori Sato, PhD^2^, Akihiko Okamura, MD, PhD^4^, Hirofumi Kawakubo, MD, PhD^1^, Manabu Muto, MD, PhD^5^, Yoshihiro Kakeji, MD, PhD^6^, Koji Kono, MD, PhD^7^, Hiroya Takeuchi, MD, PhD^8^, Masayuki Watanabe, MD, PhD^3^, Yuichiro Doki, MD, PhD^9^, Takeo Bamba, MD, PhD^10^, Takashi Fukuda, MD, PhD^11^, Hitoshi Fujiwara, MD, PhD^12^, Shinsuke Sato, MD, PhD^13^, Kazuhiro Noma, MD, PhD^14^, Hiroshi Miyata, MD, PhD^15^, Takeo Fujita, MD, PhD, FACS^16^, Yuko Kitagawa, MD, PhD, FACS^1^

1. Department of Surgery, Keio University School of Medicine, Tokyo, Japan
2. Department of Preventive Medicine and Public Health, Keio University School of Medicine, Tokyo, Japan
3. Biostatistics Unit, Clinical and Translational Research Center, Keio University Hospital, Tokyo, Japan
4. Department of Gastroenterological Surgery, Cancer Institute Hospital of Japanese Foundation for Cancer Research, Tokyo, Japan
5. Department of Therapeutic Oncology, Graduate School of Medicine, Kyoto University, Kyoto, Japan
6. Division of Gastrointestinal Surgery, Department of Surgery, Kobe University Graduate School of Medicine, Hyogo, Japan
7. Department of Gastrointestinal Tract Surgery, Fukushima Medical University, Fukushima, Japan
8. Department of Surgery, Hamamatsu University School of Medicine, Shizuoka, Japan
9. Department of Gastroenterological Surgery, Osaka University Graduate School of Medicine, Osaka, Japan
10. Department of Digestive Surgery, Niigata Cancer Center Hospital, Niigata, Japan
11. Department of Gastrointestinal Surgery, Saitama Cancer Center, Saitama, Japan
12. Division of Digestive Surgery, Department of Surgery, Kyoto Prefectural University of Medicine, Kyoto, Japan
13. Department of Gastroenterological Surgery, Shizuoka General Hospital, Shizuoka, Japan
14. Department of Gastroenterological Surgery, Okayama University Graduate School of Medicine, Dentistry, and Pharmaceutical Sciences, Okayama, Japan
15. Department of Digestive Surgery, Osaka International Cancer Institute, Osaka, Japan
16. Department of Esophageal Surgery, National Cancer Center Hospital East, Chiba, Japan

*Corresponding Author: Satoru Matsuda, MD, PhD

Department of Surgery, Keio University School of Medicine, 35 Shinanomachi, Shinjuku-ku, Tokyo, 160-8582, Japan

Email: s.matsuda.a8@keio.jp

Phone: +81-3-5363-3802, Fax: +81-3-3355-4707

**Supplementary Materials - Index**

| **Supplementary Figures and Tables** |  |
| --- | --- |
| Table S1 | *pag. 2* |
| Table S2 | *pag. 3* |
| Figure S1 | *pag. 4* |

**Table S1. Kendall’s Tau between RFS and OS (Subgroup analysis of adjuvant chemotherapy)**

| **Subgroup** | **n** | **Method** | **Kendall's Tau** | **95% CI** |
| --- | --- | --- | --- | --- |
| AC | 398 (13.2%) | Illness-death model | 0.721 | 0.667 – 0.775 |
| Non-AC | 2624 (86.8%) |  | 0.808 | 0.792 – 0.825 |
| Note: There were 132 patients in whom it was unknown whether AC was performed, but these were excluded from the analysis. Abbreviations: AC, adjuvant chemotherapy; CI, confidence interval; OS, overall survival; RFS, Recurrence-free survival. | | | | |

**Table S2. Kendall’s Tau and C-index between short-term endpoints and OS**

| **Variable** | | **HR** | **95% CI** | ***P* value** | ***C*-index** | **95% CI** |
| --- | --- | --- | --- | --- | --- | --- |
| pCR | Non-pCR | Ref |  |  | 0.521 | 0.516 - 0.527 |
|  | pCR | 0.268 | 0.179 - 0.401 | <0.001 |  |  |
| pGrade | 0 | Ref |  |  | 0.575 | 0.559 - 0.590 |
|  | 1a | 0.860 | 0.723 - 1.023 | 0.088 |  |  |
|  | 1b | 0.698 | 0.566 - 0.86 | 0.001 |  |  |
|  | 2 | 0.427 | 0.342 - 0.534 | <0.001 |  |  |
|  | 3 | 0.263 | 0.187 - 0.371 | <0.001 |  |  |
| Abbreviations: CI, confidence interval; pCR, pathological complete response; HR, hazard ratio. | | | | | | |

**Figure S1. Flowchart of the study**

Abbreviations: CF, cisplatin and 5-fluorouracil; DCF, docetaxel, cisplatin and 5-fluorouracil; NAC, neoadjuvant chemotherapy; NACRT, neoadjuvant chemoradiotherapy.
